# Variable selection methods for developing a biomarker panel for prediction of dengue hemorrhagic fever

**DOI:** 10.1186/1756-0500-6-365

**Published:** 2013-09-11

**Authors:** Hyunsu Ju, Allan R Brasier

**Affiliations:** 1 Departments of Preventive Medicine and Community Health, University of Texas Medical Branch (UTMB), Galveston, TX, USA; 2 Sealy Center for Molecular Medicine, UTMB, Galveston, TX, USA; 3 Institute for Translational Sciences, UTMB, Galveston, TX, USA

**Keywords:** Variable selection, Classification, Bootstrap sampling, Data mining

## Abstract

**Background:**

The choice of selection methods to identify important variables for binary classification modeling is critical to produce stable models that are interpretable, that generate accurate predictions and have minimum bias. This work is motivated by data on clinical and laboratory features of severe dengue infections (dengue hemorrhagic fever, DHF) obtained from 51 individuals enrolled in a prospective observational study of acute human dengue infections.

**Results:**

We carry out a comprehensive performance comparison using several classification models for DHF over the dengue data set. We compared variable selection results by Multivariate Adaptive Regression Splines, Learning Ensemble, Random Forest, Bayesian Moving Averaging, Stochastic Search Variable Selection, and Generalized Regularized Logistics Regression. Model averaging methods (bagging, boosting and ensemble learners) have higher accuracy, but the generalized regularized regression model has the highest predictive power because the linearity assumptions of candidate predictors are strongly satisfied via deviance chi-square testing procedures. Bootstrapping applications for evaluating predictive regression coefficients in regularized regression model are performed.

**Conclusions:**

Feature reduction methods introduce inherent biases and therefore are data-type dependent. We propose that these limitations can be overcome using an exhaustive approach for searching feature space. Using this approach, our results suggest that IL-10, platelet and lymphocyte counts are the major features for predicting dengue DHF on the basis of blood chemistries and cytokine measurements.

## Background

Analysis of high-dimensional data, where the number of predictors exceeds the sample size, poses many challenges for statisticians and calls for new statistical methodologies in order to select relevant variables that are correlated with one-another. In these data, feature selection is used to overcome the curse of dimensionality by removing non-essential variables to achieve a model with predictive accuracy. Consequently, the choice of a variable selection procedure becomes very important for the ability to generate reproducible findings and generalizable conclusions. In high-dimensional data it is desirable to have parsimonious or sparse representations of prediction models. Since highly complex models will be penalized by increased total error, regularization can help control the complexity of a classification by minimizing over-fitting of the training data. This approach is evaluated by maximizing goodness-of-fit and simultaneously minimizing the number of variables selected. An alternative to regularization is to develop a classifier via exploring information from many models built on a perturbed version of a learning ensemble, such as a bagging and a boosting algorithm. We evaluated different models by randomly selecting and withholding the training data to be used later for testing. The receiver characteristic operating curve is a measure by which to compare prediction accuracy based on sensitivity and specificity for both training and test data.

This study is motivated by our previous work on the identification of biomarker panels associated with dengue hemorrhagic fever [1,2]. Dengue is an acute viral infection hyperendemic to the tropics for which significant numbers are at risk. A subgroup of patients acutely infected with dengue is at risk for a later manifestation of capillary leak and hemorrhage, known as dengue hemorrhagic fever (DHF). In this study discriminative features were identified that associated with dengue hemorrhagic fever. Various blood chemistries and cytokine measurements can be associated with disease outcome. However, many of these variables are highly correlated and which of the factors will result in the most stable classifier is not known. Here we seek to extend this work by comparing the effects of various feature reduction methods to identify the most robust features for early detection of DHF.

### Study population

Active surveillance for people with dengue infection was conducted in Maracay, Venezuela. Fifty one febrile subjects with signs and symptoms consistent with dengue infection who presented at participating clinics and hospitals, or who were identified by community-based active surveillance, were included in the study. On the day of presentation, a blood sample was collected for dengue virus reverse transcription-polymerase chain reaction (RT-PCR) confirmation and clinical testing. Using the 2009 World Health Organization (WHO) criteria, 13 subjects developed DHF (dengue with warning signs; of these three were classified as severe dengue caused by plasma leakage and severe bleeding). Predictive modeling was performed using laboratory values of 51 individuals (38 DF and 13 DHF) obtained on initial presentation via binary classification models.

## Methods

### Feature selection methods identify the subset of differentially-expressed predictors that are useful and relevant in distinguishing different classes of samples. Feature reduction using Significance Analysis of Microarray (SAM)

We used Akaike information criterion (AIC) for SAM modeling. AIC is given by

(1) $$\mathrm{AIC}=‒2\ln\left(L\right)+2\left(p+1\right),$$

where L is the maximum likelihood for the model, and p is number of covariates estimated in the model [3].

Bayesian information criterion (BIC) is given by

(2) $$\mathrm{BIC}=‒2\ln\left(L\right)+\ln\left(n\right)\times \left(p+1\right),$$

where n is the samples size, and p are defined as those variables in AIC [4].

### Learning ensemble

For the Learning Ensemble, we used a stochastic gradient boosted model (TreeNet). TreeNet is a generalized tree boosting algorithm that is an accurate and effective off-the-shelf procedure for data mining [5,6]. Software implementation of the bagging ensemble model was from Salford Predictive Modeler version 7.0 from Salford Systems.

### Bayesian model averaging

Bayesian model averaging (BMA) was used to consider all the possible 2^p^ model configurations from the model space as the potentially correct mode. BMA was applied using the R library BMA (http://cran.r-project.org).

### Stochastic Search Variable Selection (SSVS)

The SSVS models were performed using WinBUGS (http://www.mrc-bsu.cam.ac.uk/bugs/winbugs). The models can fit with the R interface in the WinBUGS, R library R2WinBUGS (http://cran.r-project.org).

### Generalized Path Seeker (GPS) modeling

Logistic regression models are estimated using the maximum likelihood method. Logistic modeling has a binary response y_i_ ∈ {0, 1}, and assuming

(3) $$\mathrm{Pr}\left(y=1|x\right)=1/\left(1+\exp\left(‒x^{T}\beta \right)\right).$$

Automated stepwise logistic regression model selection has the problem of instability and overfitting of subset selection in multivariate regression by retaining redundant information and noisy variables. The method sequentially drops and adds predictor variables by examining the mean squared error or a modified version of it. Recent techniques, such as shrinkage and regularized estimation, can account for and correct the overfitting. We used the optimized L1 and L2 regularization generalized linear model to select to most important features. Regularized and shrinkage estimation methods such as a lasso (least absolute shrinkage and selection operator) estimator help address variable selection and multicollinearity [7]. The lasso utilizes the L1 penalty and does both continuous shrinkage and automated variable selection simultaneously. For a binary response variable and the logistic regression models, the lasso estimator is estimated by penalized the negative log-likelihood with the L1-norm. The penalty term is chosen by a cross-validation technique to evaluate the out-of-sample negative log-likelihood. The elastic net combines the L1 and L2 penalizing terms and possesses a grouping effect, i.e., in a set of variables which have high pairwise correlations, the elastic net groups the correlated variables together [8].

The coefficient vector β that minimizes the penalized log-likelihood,

(4) $$\begin{array}{ll} \beta^{\wedge }=\mathrm{argmin\beta}\in \mathrm{Rp}\;‒\sum & \left(\mathrm{yi}\;\log\;\mathrm{pi}+\left(1‒\mathrm{yi}\right)\right)\\ & \left(\log\left(\left(1‒\mathrm{pi}\right)\right)+\lambda_{1}||\beta |{|}_{1}+\lambda_{2}||\beta |{|}_{2},\right) \end{array}$$

where p_i_ = Pr(y = 1|x).

We used a soft loss function which is robust to the influence of outliers. Resampling techniques and cross-validations were evaluated to reduce variability in the prediction measures. Bootstrap methods are introduced when an averaged prediction is made using multiple models generated on random resamples of the observations with replacement. Bootstrap resampling started with fitting the logistic model in a bootstrap sample of n subjects, which was drawn with replacements from the original sample. Zou (2006) proposed the adaptive lasso, which permits different weights for different parameters, and is also shown to have the oracle property [9]. The comparisons of penalized regression methods in binary response and logistic regression such as the ridge, lasso, and elastic net were conducted. To estimate the coefficient, we performed generalized path seeker (GPS), a high speed lasso-style regression from Friedman (2012) to regularize regression [10]. GPS demonstrates the regularized regression based on the generalized elastic net family of penalties. Software implementation of GPS is available in Salford Predictive Modeler version 7.0 from Salford Systems.

### Multivariate Adaptive Regression Splines: MARS

MARS basis functions are combined as a weighted sum of

(5) $${\hat{y}}_{i}=a_{0}+\sum_{k=1}^{p}a_{k}B_{k}\left(x\right),$$

where yˆ is the response described by the model, a_0_ the coefficient of the constant basis function (intercept), p the total number of basis functions and ak the coefficient of the kth basis function B_k_(x). MARS models use hockey stick basis functions of the form (x − t)_+_ and (t − x)_+_, with t being the knot. The basis functions in MARS are single truncated spline functions or a product of two or more spline functions for different predictors. The first order MARS model was built without interactions to over-fit the training data. A maximum number of basis functions equal to 30 was used as the stopping criterion. The model was pruned using a ten-fold and generalized cross validation. The optimal model was selected based on evaluation of the model complexity and its predictive quantities for the test sets. Software implementation of MARS model is available in Salford Predictive Modeler version 7.0 from Salford Systems.

### Random Forest (RF)

RF modifies the classic CART (Classification and regression tree) algorithm by randomly selecting from a subset of descriptors, rather than choosing the best split among all samples. This procedure is repeated until a sufficiently large number of trees have been computed. RF performs a bootstrapping cross-validation procedure in parallel with the training step of its algorithm. This method allows some of the data to be left out at each step, then used later to estimate the accuracy of the classifier after each instance has been carried out. Software implementation of the RF model is available in Salford Predictive Modeler version 7.0 from Salford Systems.

### Nonlinear testing procedures

Modeling was performed to assess the linear or non-linear association of binary responses variables of selected variables. For each part predictor, we also examined the log-likelihood ratio test p-values comparing the deviance between the full model and the model without that variable. We calculated the projection (hat) matrix, Cook’s distance, various residuals and the estimated probabilities versus each predictor to evaluate outliers and identify influential points in the models. We used both the change in residual deviance (as in parametric or nonparametric models), and the area under the receiver operating characteristic curve (ROC) to compare of the performance of the statistical models. The area under the curve measures indicate the ability of the model to discriminate between the outcome groups. A score of 0.5 indicates that a model has no discriminatory ability and a score of 1 indicates the two groups are perfectly discriminated.

## Results and discussion

In this study, we measured various blood chemistries and cytokine measurements that are associated with disease outcome. We next sought to identify those variables that were most informative for outcome. To improve the model performance, we sought to first reduce the dimensionality. Feature reduction removes meaningless features which are not related to a studied disease, thus leading to overfitting of a classifier in high dimensional data. This happens because without prior removal of irrelevant genes, the classification problem is known as the small sample size problem (the number of features far exceeds the number of samples in data set). Dimensionality can be reduced by selecting an appropriate subset among existing variables. For this purpose, we applied two sample t-test or where appropriate, a two-sample t-test on log transformed data. We addressed the multiple testing using false discovery rate (FDR), a re-sampling method have an improved ability to reject a false hypothesis compared to the Bonferroni correction.

To accomplish this, we performed variable selection using a SAM test. SAM is a widely used permutation-based approach to identifying differentially expressed genes when assessing statistical significance using FDR adjustment in high dimensional datasets [11]. Efron, Tibshirani, Storey et al. (2001) developed an empirical bayesian approach using non-informative priors and deriving the posterior probability difference for each predictors without having to run t tests or Wilcoxon tests to identify which were differentially expressed [12]. In some cases, a heuristic approach is investigated for feature selection by integrating correlation, histogram, and other statistical measures. We used the AIC information criterion on the modeling (Methods) with the false discovery adjustment to select the top 25 variables with the most significant p-values, based on the training data for each fold of a 10-fold cross validation. Using these variables, the training data were split into two parts: one to fit penalized logistic models and one to select tuning parameters.

**Figure 1:**
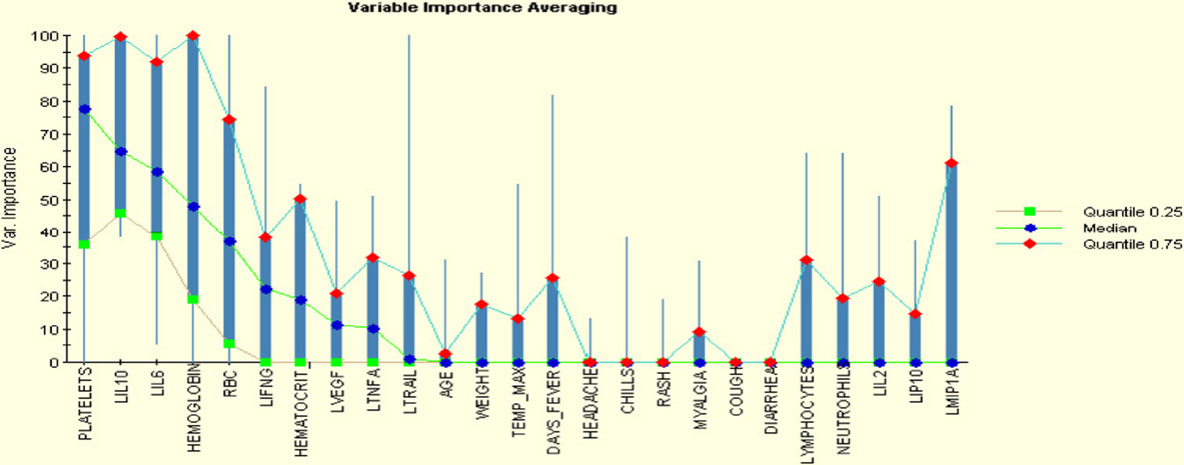
**Learning Ensemble Variable Importance.** A Shown is a rank ordered list of the 25 variables selected by SAM using t-test and corrected by FDR. For each is shown the importance for each variable, estimated as its frequency of appearance in the bootstrap aggregating tree.

**Figure 2:**
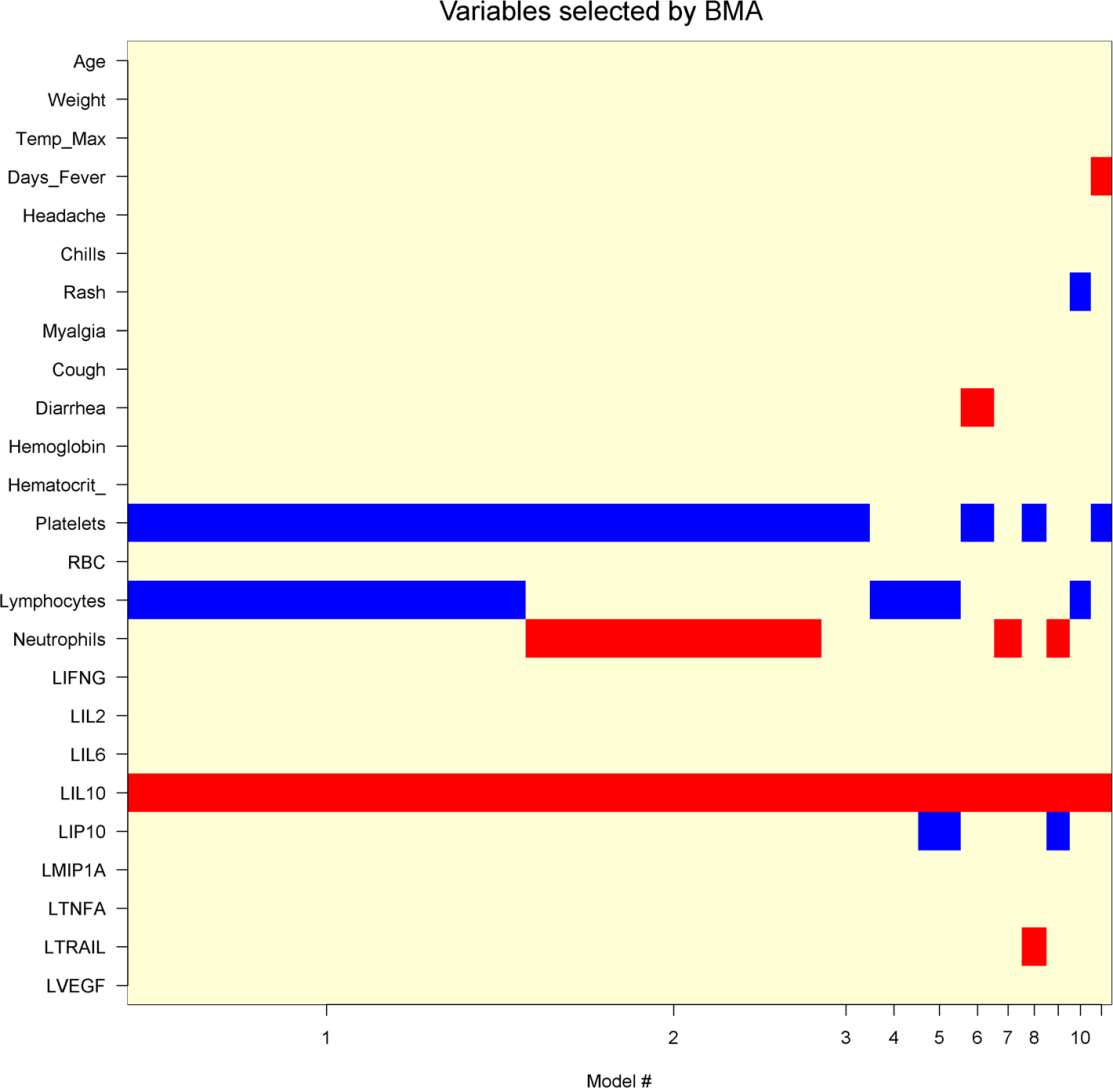
**Bayesian Model Averaging.** The X axis represents the models with the highest posterior probability and the Y-axis represents the variable included in the model, where the red (positive) or blue (negative) blocks indicate significant regression coefficients. Note that IL10, lymphocytes and platelets appear in the model with the highest posterior probability.

We next applied different feature selection methods to identify the most informative predictive variables. First we examined variables identified by learning ensemble. Learning ensemble approach was developed by Breiman (1996), who adopted the bootstrap sampling to generate different base leaner classifiers [13]. The bagging (bootstrap and aggregation) ensemble is the most popular strategy for aggregating the output of the base learning, i.e., voting for classification. The bagged ensemble method can be used with unstable classifiers to reduce the large variance effect. Bagging often outperforms the base learning algorithm and improves the performance of the model. The important variables identified by learning ensemble were Platelets, IL-10, and IL-6 (Figure 1). Each of these have > 50 average values of appearing in the aggregating tree. The training ROC measure of the bagging ensemble is 0.994, and the overall average prediction accuracy is 80% (Table 1). Second, Bayesian approaches were evaluated. Bayesian model averaging (BMA) is an approach to assess the robustness of results in terms of alternatives by calculating posterior distribution over coefficients and models. BMA produces a posterior probability for each possible model in addition to one for each predictor [14]. Using BMA, model uncertainty can be incorporated into conclusions about parameters and predictions. The BMA analysis identified three variables-IL-10, platelets, and lymphocytes-have a high probability of predicting hemorrhagic fever (Figure 2). Finally, stochastic search variable selection (SSVS) was proposed by George and MaCulloch (1993) for linear models [15] and Albert and Chib (1993) for binary and polychotomous outcome models [16]. The Gibbs sampler method for SSVS allows interrogation of the model space efficiently without fitting all possible models and not having the inferences driven by the model assumptions. The Bayesian hierarchical methods of variable selection and classification are complimentary approaches to high dimensional data in that the uncertainty in the model choice can be incorporated into the analysis. For this analysis, 25,000 iterations of the Markov Chain Monte Carlo (MCMC) were completed by removing the first 1000 for burn-in and saving every 5th iteration to maximize storage. In the case of a binary outcome, the latent probit model in which posteriors distribution for the latent indicator variables are estimated via MCMC could be used. Similarly, the SSVS models ranked the following as importance predictors: diarrhea, IL6, platelets, IL-10, lymphocytes, and trail (data not shown).

**Table 1:** Model assessment of the predictive power

**Predictive**	**Accuracy**	**Accuracy**	**Accuracy**	**AUC**	**AUC**	**AUC**
**Models**	**Train**	**Test**	**∆**	**Train**	**Test**	**∆**
Bagging Ensemble	0.804	0.745	0.059	0.994	0.704	0.29
GPS	0.882	0.804	0.078	0.976	0.921	0.055
MARS	0.902	0.726	0.176	0.955	0.789	0.166
TreeNet Gradient Boosting	0.804	0.745	0.059	0.994	0.704	0.29

Shown are the predictive powers for training and test samples based on a 10-fold cross validation procedure.

∆, difference between the training sample and the test sample; AUC, area under the curve.

**Figure 3:**
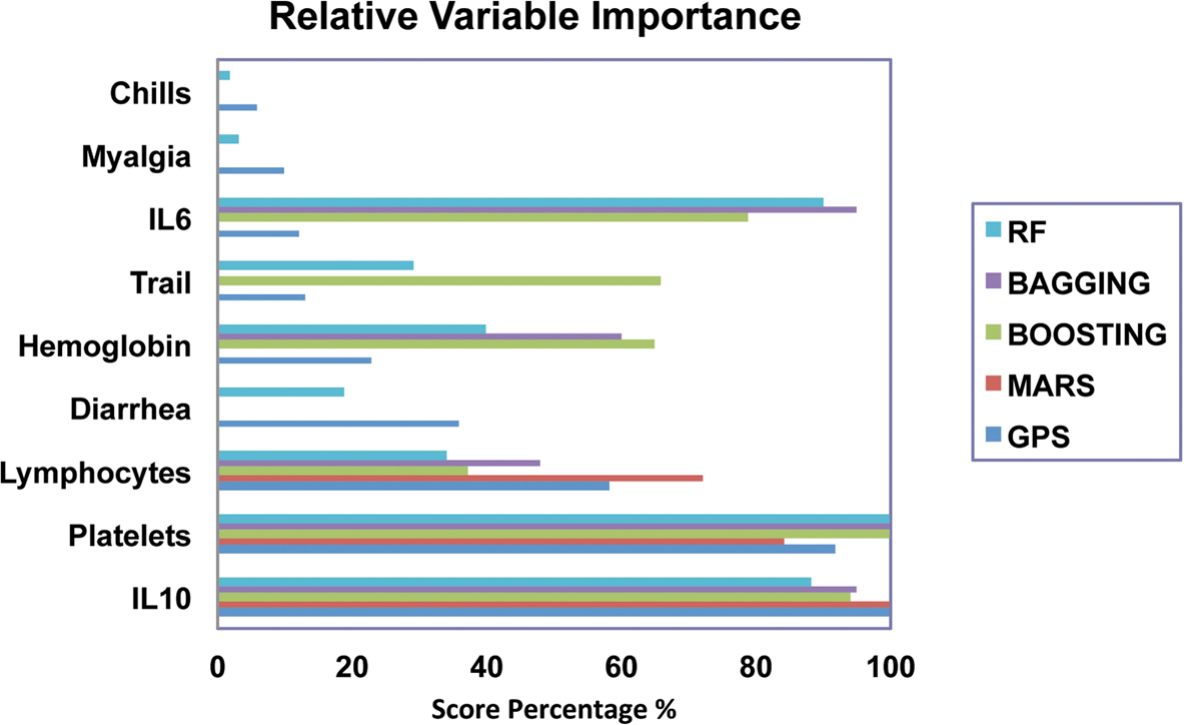
**Relative Variable Importance.** Shown is relative variable importance for GPS, MARS, Boosting, Bagging and RF models. The BMA/SSVS relative variable importance cannot be calculated and is not shown.

Next we identified variables using five different feature reduction classification methods, including generalized path seeker (GPS), multivariate adaptive regression splines (MARS), TreeNet, Boosting, and Random Forest (RF) (Figure 3). The GPS is a high speed lasso-style logistic regression from Friedman (2012) to regularize regression [10]. The MARS method of Friedman (1991) is a nonparametric regression method that estimates complex nonlinear relationship by a series of truncated spline functions of the predictors [17]. The random forest (RF) classifier of Breiman (2001) generates a random decision tree by selecting a feature subset randomly at each node, but then performing the conventional split selection within the selected feature [18]. The randomization procedure is introduced only into the feature selection, not into the choice of split points on the selected feature subset.

The relative variable importance for the MARS model and the basis functions are shown in Table 2. Here, IL-10 was the most important variable (100% relative importance), followed by lymphocytes (84% importance) and platelets (72%). Similarly, relative importance for the variables appearing in the GPS model are shown in Table 3, where the same rank order of importance, IL-10 (100%), lymphocytes (92%) and platelets (58%) was observed.

**Table 2:** MARS Basis Functions

**B**_**m**_	**Definition**	**am**	**Variable**	**Variable**
			**descriptor**	**Importance**
BF1	(IL10 − 5.17)_+_	0.312	IL10	100%
BF3	(Lymphocytes − 8)_+_	−0.011	Lymphocytes	84%
BF4	(Platelets − 45)_+_	−0.004	Platelets	72%

Shown are the basis functions (BF) for the MARS model for Dengue hemorrhagic fever.

B_m_ each indiviual basis function, a_m_, coefficient of the basis function. (y)_+_, = max(0, y).

**Table 3:** GPS- generalized lasso estimators

**Variable**	**Coefficients**	**Variable Importance**
IL10	0.611	100%
Lymphocytes	−0.025	92%
Platelets	−0.042	58%

Shown are the coefficients for the generalized lasso estimators and relative importance.

**Figure 4:**
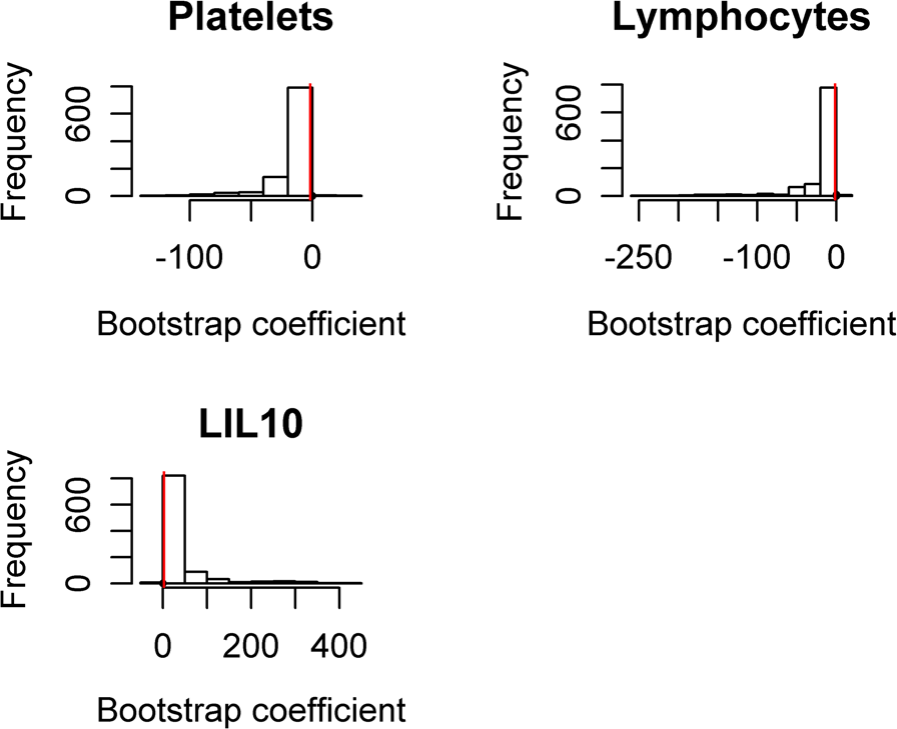
**Bootstrap Coefficients.** For each variable shown, the frequency of coefficient is plotted for 999 bootstrap estimations. Note that the coefficients in the GPS model (Table 1) fall within the 95% confidence limits of the bootstrap estimation.

**Figure 5:**
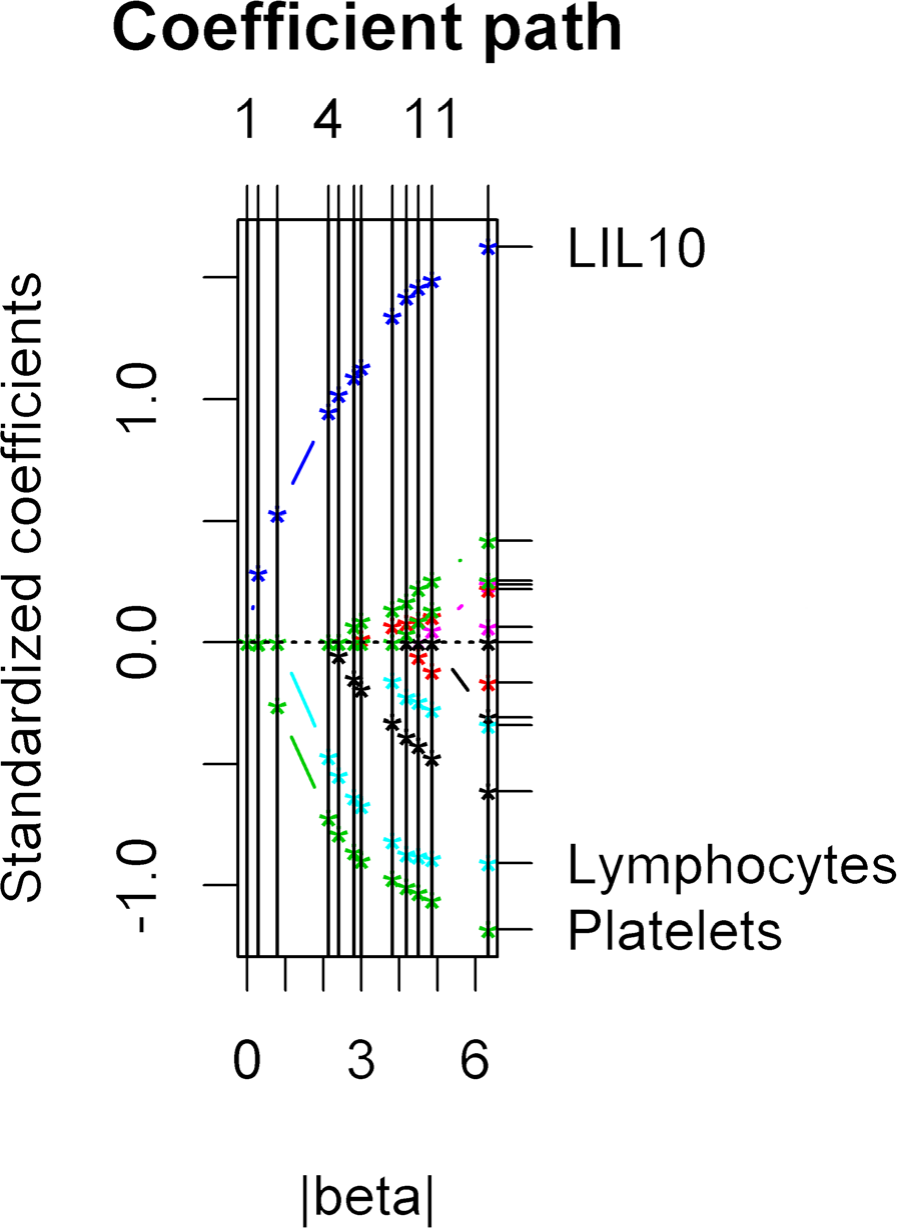
**Regularized Regression Path.** Shown is the solution path β(s) as a function of s. As we can see, any segment between two adjacent vertical lines is linear, hence the whole solution path is piecewise linear. Another important feature of this solution path is, over a certain range of values of s, only the relevant predictor variables, that is, IL10, lymphocytes, and platelets have nonzero fitted coefficients

Overall, this comparison indicated that IL-10, platelet count and lymphocytes were the three most important variables that consistently shown high relative importance across all models. We also evaluated the nonlinearity of these important predictors

(IL-10, platelets, and lymphocytes) using the deviance testing procedure (χ^2^ statistics = 16.564, p-value is 0.06) using generalized additive modeling (GAM) in a manner similar to that earlier shown by Brasier et al. [2]. GAMS is a diagnostic graphical tool to evaluate e the partial residual plot r identifying nonlinear relationship between the response and covariates for generalized additive models [19]. The nonlinearity of the three predictor variables was relatively unimportant. For the bootstrap estimators, we further examined the model coefficients using resampling techniques. In this analysis, B = 999 rounds of bootstrapping were performed. For each variable, the model coefficient fell within the bootstrap-estimated 95% confidence interval (red vertical line, Figure 4). These findings indicate that the coefficient estimates appear stable. The solution path β(s) as a function of s was shown (Figure 5). Any segment between two adjacent vertical lines is linear, hence the whole solution path is piecewise linear. The indices of predictor variables are labeled on the right side axis. Predictors IL10, Lymphocytes, and Platelets are relevant variables. As we can see, over a range of s, only these three predictors have nonzero fitted coefficients.

Analysis of the model performance on the training data set using ROC measures indicated that the GPS model had an AUC of 0.92, the MARS model had an AUC of 0.79, and the TreeNet model had an AUC of 0.70 (Table 1).

Based on this ROC comparison, we concluded that the GPS model has the best prediction accuracy of the models tested. Another comparison of the model performance can be based on whether it will generalize well. In this analysis, we estimated the change in AUC in the test set relative to the training set. Here, the GPS model had the smallest difference in AUC (Table 1), suggesting that it will generalize better than the other models.

## Conclusions

In this article, we compared several statistical methods for feature reduction and constructing a predictive modeling of developing dengue hemorrhagic fever disease. Our attempts to identify the most informative predictors is based on the assumption that different variable reduction techniques have inherent biases, resulting in the omission of potentially informative predictors and inclusion of uninformative ones. For example, the learning ensemble did not identify lymphocytes as an important variable, yet this variable was identified by the BMA methods. Model comparison suggests that GPS method with IL10, Lymphocytes, and Platelets outperforms other models tested on the basis of AUC and predicted ability to generalize. We recognize that our study is limited because of the small data set. Further evaluation of this modeling procedure on a large independent data set is needed. It also will be a meaningful study to explore the effect of sample size on the performance of various methods.

## Availability of data

Data are available on the NIAID Clinical Proteomics Center Web site at:

https://bioinfo.utmb.edu/CPC/Projects/default.jsp

## Competing interests

The authors declare that they have no competing interests.

## Authors’ contributions

HJ was involved in research concept and design, collection and/or assembly of data, data analysis and interpretation and writing the article. AB was involved in research design, data interpretation and helped to write and review this work. All authors read and approved the final manuscript.
